# Evaluating the Long-Term Utility and Cost-Effectiveness of Computed Tomography (CT) Surveillance Beyond Five Years After Radical Cystectomy: A 10-Year Follow-Up Study

**DOI:** 10.7759/cureus.92501

**Published:** 2025-09-16

**Authors:** Omar Elsandoby, Rahaf Akroush, Alison Roodhouse, Venkata Ramana Murthy Kusuma, Matthew Perry

**Affiliations:** 1 Urology, Royal Surrey County Hospital, Guildford, GBR

**Keywords:** bladder cancer, cost-effectiveness, ct surveillance, follow-up, radical cystectomy

## Abstract

Introduction

While computed tomography (CT) imaging is crucial for the early detection of recurrence after radical cystectomy, the clinical value and cost-effectiveness of routine surveillance beyond five years remain unclear. This study evaluates the diagnostic yield and economic justification of CT scans performed at seven and 10 years postoperatively.

Methodology

This retrospective study included 46 patients who underwent radical cystectomy for bladder cancer in 2013-2014, ensuring a minimum follow-up of 10 years. Clinical and pathological variables were analysed for correlation with recurrence or metastasis. The diagnostic utility of CT scans at seven and 10 years was assessed. Statistical analysis included the chi-squared test, Mann-Whitney U test, and logistic regression using IBM SPSS Statistics for Windows, V. 27.0 (IBM Corp., Armonk, NY, USA).

Results

At 10 years, 19 patients (41.3%) were alive. All metastases (n=14) occurred within the first two years post-surgery; no positive findings were detected on CT scans at seven or 10 years. Metastasis was significantly associated with the presence of carcinoma in situ (CIS) (p=0.02), positive surgical margins (p=0.004), and fewer dissected lymph nodes (p<0.001). Logistic regression identified positive margins as an independent predictor of metastasis (OR: 9.33; p=0.008).

Conclusion

Routine CT surveillance beyond five years post-cystectomy shows minimal clinical benefit in unselected patients. A risk-adapted approach, focusing on pathological factors such as surgical margin status and CIS, may optimize follow-up strategies and resource utilization.

## Introduction

Computed tomography (CT) has become a cornerstone of diagnostic medicine, providing precise and rapid imaging that enhances the detection, monitoring, and evaluation of a wide range of conditions. In urologic oncology, particularly bladder cancer, CT imaging is integral to postoperative surveillance following radical cystectomy. While early follow-up scans are crucial for detecting recurrence or metastasis, the long-term value of routine imaging, especially beyond five years after surgery, remains uncertain [1,2].

Radical cystectomy, the standard treatment for muscle-invasive bladder cancer, carries a substantial risk of recurrence, most often within the first two to three years postoperatively [1,3]. Nevertheless, late recurrences do occur [4], raising the question of whether extended imaging surveillance is justified. Prolonged follow-up must balance potential clinical benefits with the risks and costs of continued imaging, including radiation exposure, false positives, incidental findings, and patient anxiety [5,6].

As healthcare systems move towards value-based care, the cost-effectiveness of CT surveillance beyond five years assumes greater importance. This study aims to assess the clinical utility and economic impact of CT imaging performed at seven and 10 years after radical cystectomy, with the goal of informing future follow-up guidelines and optimizing long-term surveillance strategies in bladder cancer [5-7].

## Materials and methods

This retrospective study included all patients who underwent radical cystectomy for bladder cancer in 2013 and 2014, providing a minimum follow-up duration of 10 years (a total of 46). We excluded patients who had a cystectomy for non-cancer reasons. The primary objective was to evaluate the cost-effectiveness of follow-up CT scans performed after five years post-surgery, i.e., at seven and 10 years. 

Demographic and clinical data were extracted and analysed to assess trends and outcomes. Variables included age, gender, American Society of Anesthesiologists (ASA) score, and World Health Organization (WHO) performance status. All radiological and histopathological results data were collected retrospectively. A correlation analysis was performed to examine the relationship between recurrence or metastasis and key oncologic and treatment factors: primary stage, presence of carcinoma in situ (CIS), primary tumour grade, administration of neoadjuvant chemotherapy, final pathological stage and grade, margin status, lymph node involvement, and receipt of adjuvant therapy.

Survival analysis was conducted to estimate cancer-specific survival and overall survival rates at both the seven- and 10-year marks. Additionally, the clinical utility of CT imaging at these time points was assessed by determining the percentage of scans that yielded positive findings related to disease recurrence or progression. Scans were typically assessed by a dedicated uro-radiology specialist. 

No missing data were encountered in the included patients. Data were analysed using IBM SPSS Statistics for Windows, V. 27.0 (IBM Corp., Armonk, NY, USA). Numerical data were described as mean, standard deviation, and range. Categorical data were described as numbers and percentages. Numerical variables were explored for normality using the Kolmogorov-Smirnov test and the Shapiro-Wilk test. Comparisons between metastatic and non-metastatic were done using Student's t-test for normally distributed data and the Mann-Whitney U test for non-normally distributed data. Comparisons between categorical variables were performed using the chi-squared test or Fisher's exact test as appropriate. Stepwise logistic regression was done to give adjusted odds ratio (OR) and measure the magnitude of the effect of different factors for the development of metastasis. A p-value of less than 0.05 was considered statistically significant. All tests were two-tailed.

## Results

A total of 46 patients who underwent radical cystectomy in 2013 and 2014 were included in this study, providing a minimum follow-up duration of 10 years. The demographic data of the patients is as follows: the mean age was 71.4±8.7 years, with a range of 47-85 years. The rest of the demographic data is shown in Table 1.

**Table 1:** Patients' demographic data. ASA: American Society of Anesthesiologists; WHO: World Health Organization

		No.	%
Gender	Female	8	17.4
	Male	38	82.6
ASA	I	14	30.4
	II	24	52.2
	III	7	15.2
	IV	1	2.2
WHO	0	17	37
	1	29	63

Regarding the preoperative staging of the included patients, five patients (11.4%) had pathological tumour stage a (pTa) disease, nine patients (20.5%) had pT1, 24 patients (54.5%) had pT2, one patient (2.3%) had pT3, and five patients (11.4%) had pT4. Concomitant CIS was present in eight patients (17.4%). In terms of tumour grading, 40 patients (87%) had G3 disease, while six patients (13%) had G2 disease. Four patients (8.7%) received intravesical mitomycin C (MMC), and 15 patients (32.6%) received intravesical Bacillus Calmette-Guérin (BCG). Nineteen patients (41.3%) underwent neoadjuvant chemotherapy.

All patients had robot-assisted radical cystectomy (RARC). One procedure was abandoned due to irresectable locally advanced disease. An extended template for lymph node dissection was performed in 38 patients (82.6%). With regard to urinary diversion, 42 patients (91.3%) had an ileal conduit, and four patients (8.7%) had a neobladder.

The final histological examination revealed that six patients had pathological tumour stage 0 (pT0) disease, two had pTa, two had pT1, three had pT2, eight had pT3, and 12 had pT4. CIS was found in 14 patients (31.1%). Nine patients (20.9%) had positive surgical margins, and six patients had positive lymph nodes. Three patients received adjuvant treatment following cystectomy.

At the 10-year follow-up, 19 patients (41.3%) were still alive. Twenty-four patients (52.2%) died before reaching the seven-year mark, and an additional six patients (6.5%) died between years 7 and 10. The causes of death included metastasis from the bladder in 14 patients (53.8%), while other causes were pulmonary embolism (PE), COVID-19, dementia, head injury, leukemia, myocardial infarction (MI), pancreatic cancer, peritonitis, pulmonary fibrosis, local recurrence, renal failure, and urosepsis. Notably, all patients who developed metastasis did so within the first two years after surgery.

When evaluating the diagnostic yield of CT scans, 21 patients underwent imaging at seven years post-cystectomy, and none showed positive findings. Similarly, 19 patients had CT scans at the 10-year mark, which also revealed no positive findings.

Correlation analysis between metastasis and various variables revealed statistically significant associations with the presence of CIS in the final histology (p=0.02) and positive surgical margins (p=0.004), as detailed in Table 2.

**Table 2:** Correlation analysis between metastasis and different variables. ASA: American Society of Anesthesiologists; WHO: World Health Organization; CIS: carcinoma in situ; H/O of MMC: history of myelomeningocele; H/O of BCG: history of Bacillus Calmette-Guérin; Mets.: metastasis; LND: lymph node dissection; PT: pathological tumour stage

		Mets.		Test	
		No (n (%))	Yes (n (%))	Value	P-value
Age (years)	Mean±SD	71.5±7.3	71.2±11.6	t=0.112	0.911
	Range	52-82	47-85		
Gender	Female	6 (75)	2 (25)	χ^2^=0.135	0.713
	Male	26 (68.4)	12 (31.6)		
ASA	I	10 (71.4)	4 (28.6)	NA	NA
	II	18 (75)	6 (25)		
	III	4 (57.1)	3 (42.9)		
	IV	0 (0)	1 (100)		
WHO	0	11 (64.7)	6 (35.3)	χ^2^=0.301	0.583
	1	21 (72.4)	8 (27.6)		
Primary CIS	No	25 (65.8)	13 (34.2)	χ^2^=1.471	0.225
	Yes	7 (87.5)	1 (12.5)		
PT	PTa	5 (100)	0 (0)	NA	NA
	PT1	6 (66.7)	3 (33.3)		
	PT2	15 (62.5)	9 (37.5)		
	PT3	1 (100)	0 (0)		
	PT4	3 (60)	2 (40)		
H/O of MMC	No	29 (69)	13 (31)	NA	1.000
	Yes	3 (75)	1 (25)		
H/O of BCG	No	21 (67.7)	10 (32.3)	χ^2^=0.149	0.699
	Yes	11 (73.3)	4 (26.7)		
Neoadjuvant chemo	No	18 (66.7)	9 (33.3)	χ^2^=0.259	0.611
	Yes	14 (73.7)	5 (26.3)		
Radicality (radical/partial)	Abandoned	0 (0)	1 (100)	NA	0.304
	Radical	32 (71.1)	13 (28.9)		
LND	No	4 (50)	4 (50)	χ^2^=1.751	0.186
	Yes	28 (73.7)	10 (26.3)		
Diversion (conduit/neo)	Conduit	29 (69)	13 (31)	NA	1.000
	Neo	3 (75)	1 (25)		
Final CIS	No	18 (58.1)	13 (41.9)	χ^2^=5.447	0.020
	Yes	13 (92.9)	1 (7.1)		
Final grade	G1	1 (50)	1 (50)	NA	NA
	G2	2 (100)	0 (0)		
	G3	20 (62.5)	12 (37.5)		
Margin	Negative	28 (82.4)	6 (17.6)	χ^2^=8.499	0.004
	Positive	3 (33.3)	6 (66.7)		
Adjuvant treatment	No	28 (75.7)	9 (24.3)	NA	NA
	Yes	3 (37.5)	5 (62.5)		

Another correlation analysis using the Mann-Whitney U test, shown in Table 3, demonstrated a statistically significant relationship between metastasis and the total number of lymph nodes dissected (p<0.001). Patients with a median total lymph node count of 11 or fewer had a higher incidence of metastasis and consequently lower survival rates.

**Table 3:** Correlation between metastasis and number of lymph nodes. Mets.: metastasis

	Mets.						Test	
	No			Yes				
	Median	Minimum	Maximum	Median	Minimum	Maximum	Value	P-value
No. of positive lymph nodes	0	0	8	0	0	6	U=161.0; z=1.39	0.390
No. of lymph nodes	16	7	33	11	0	15	U=55.0; z=-3.505	<0.001

Logistic regression analysis, presented in Table 4, identified positive surgical margins as a statistically significant predictor of metastasis (B=2.234; SE=0.838; p=0.008). The OR was 9.33 (95% CI: 1.81-48.24), indicating that patients with positive margins had 9.3 times higher odds of developing metastasis compared to those with negative margins.

**Table 4:** Multivariate analysis of margin status. A p-value of <0.05 is considered significant. B: regression coefficients; S.E.: standard error of the coefficient; OR: odds ratio; 95% CI for OR: 95% confidence interval for the OR

	B	S.E.	Wald	df	P-value	OR	95% CI for OR	
							Lower	Upper
Margin	2.234	0.838	7.103	1	0.008	9.33	1.81	48.24
Constant	-1.54	0.45	11.725	1	0.001	0.21		

## Discussion

Several studies have investigated the clinical value and economic implications of follow-up CT scans in patients with bladder cancer undergoing radical cystectomy. Consistently, the literature supports the notion that the majority of recurrences occur within the first few years following surgery and that the utility of routine imaging significantly decreases beyond the five-year mark. This aligns closely with our findings, where all patients who developed metastases did so within the first two years postoperatively and no positive findings were detected on CT scans performed at either the seven- or 10-year follow-up points.

A retrospective analysis by Boorjian et al. similarly reported that most recurrences were concentrated within the initial 2-3 years following cystectomy, with late recurrences being rare and typically associated with poor prognoses regardless of the timing of detection [8]. Our study corroborates this pattern, as late metastases beyond the two-year period were not observed, suggesting limited survival benefit from extended imaging in our patient cohort.

The financial implications of prolonged imaging have also been questioned. Kusaka et al., through a cost-effectiveness study using a Markov model, concluded that extending CT surveillance beyond five years results in disproportionately high costs with minimal benefit in terms of quality-adjusted life years (QALYs) [5]. Our data support this assessment: despite performing CT scans at the seven- and 10-year marks in a significant subset of patients, there were no positive findings, indicating low diagnostic yield and highlighting the potential for unnecessary healthcare expenditures.

Guidelines from the American Urological Association (AUA) and European Association of Urology (EAU) recommend structured post-cystectomy follow-up but acknowledge the lack of robust evidence supporting routine imaging beyond five years [9,10]. Recent literature, including a review by Kusaka et al., advocates for a risk-adapted surveillance approach, suggesting that extended imaging may only be justified in high-risk patients, that is, those with features such as lymph node involvement or positive surgical margins [5]. Our findings reinforce this risk-adapted perspective. Statistically significant correlations were identified between metastasis and the presence of CIS in the final histology, positive surgical margins, and a lower number of dissected lymph nodes. Moreover, logistic regression analysis confirmed that positive surgical margins were a strong independent predictor of metastasis, with an OR of 9.33. This underlines the importance of individualized follow-up strategies based on pathological risk factors rather than blanket imaging protocols.

Solsona et al. further examined late recurrences after radical cystectomy, concluding that while they do occur, they are infrequent and rarely curable, casting doubt on the value of continued long-term imaging. Our long-term follow-up data align with this, as the absence of metastases beyond two years post-surgery, combined with the lack of CT findings at later time points, strongly suggests limited benefit in extending routine surveillance imaging in unselected patients [11].

In summary, our study supports the growing consensus in the literature that routine CT surveillance offers its greatest value within the first few years following radical cystectomy. Beyond this window, particularly after five years, the utility diminishes considerably, especially in patients without high-risk pathological features. Our findings highlight the importance of a tailored, risk-based surveillance strategy that can optimize clinical outcomes while minimizing unnecessary interventions and associated costs. It is also important to note the radiation burden associated with repeated imaging. A single-phase CT scan delivers a median dose of approximately 15 mSv, which is equivalent to about five to six years of natural background radiation. This further underlines the need to rationalize follow-up imaging, balancing clinical benefit against potential long-term risks. The limitations of our study are the relatively small number of cases and its retrospective nature. We are planning to continue follow-up and include additional patients who underwent RARC and received CT surveillance beyond the five-year postoperative point.

## Conclusions

Our long-term follow-up study confirms that the risk of metastatic recurrence after radical cystectomy is highest within the first two postoperative years, with no new events detected beyond that timeframe. This pattern indicates that routine CT surveillance beyond five years offers minimal additional value for the majority of patients. Instead, a risk-adapted approach should be prioritised, with closer monitoring reserved for individuals with high-risk pathological features such as positive surgical margins, lymphovascular invasion, or concomitant CIS. Such tailoring would reduce unnecessary radiation exposure, false positives, and healthcare costs while ensuring that high-risk patients receive appropriate surveillance intensity. These findings support the refinement of follow-up protocols to balance clinical benefit with patient safety and resource efficiency.
